# Editorial: Updates on epigenetic regulation of endocrine disorders with polygenic traits: What is new?

**DOI:** 10.3389/fendo.2022.1073226

**Published:** 2022-11-17

**Authors:** Ahmet Uçar, Barış Binay, Bibekanand Mallick

**Affiliations:** ^1^ Department of Pediatric Endocrinology and Diabetes, University of Health Sciences, Şişli Hamidiye Etfal Education and Research Hospital, Istanbul, Turkey; ^2^ Department of Bioengineering, Gebze Technical University, Kocaeli, Turkey; ^3^ RNAi and Functional Genomics Laboratory, Department of Life Science, National Institute of Technology, Rourkela, India

**Keywords:** epigenetic, non-coding RNAs (ncRNA), methylation, obesity, diabetes, Graves disease, metabolomics, endocrine disruptors

A major impetus for a change in paradigm on the genetic approach to endocrine disorders with oligogenic/polygenic traits (influenced by two or more genes) has been the significant lack of available data regarding the frequency of pathogenic variants in coding regions of human genome (1). The common molecular changes that underlie epigenetic control are DNA methylation, chromatin remodeling, covalent histone modification, the localization of histone variants, and feedback loops (2). Epigenetic changes may be targeted to specific genes by either transcription factors (TFs) or non-coding RNAs (ncRNAs). Non-coding RNAs may be classified as small ncRNAs (including microRNAs (miRNAs), small interfering RNAs (siRNAs), small nucleolar RNAs (snoRNAs), PIWI interacting RNAs (piRNAs)) and long ncRNAs (lncRNAs) (3). It is not surprising that during the past two decades, researchers have discovered aberrations in the expression of ncRNAs associated with many human diseases such as cancer (4), cardiovascular diseases (5) and neurodegenerative disorders (6). Understanding the causal link between ncRNAs and human diseases have paved the path for novel strategies, such as the use of anti-ncRNA oligonucleotides to inhibit ncRNA for treating some types of cancer (7) and use of ncRNAs to enhance the sensitivity or reduce the resistance of the chemotherapeutic drugs (8–10).

This Research Topic is dedicated to exploring the most recent advances in evaluation of ncRNAs and the current knowledge on the putative influence of epigenetics with the evolution and phenotypic severity of endocrine disorders with oligogenic/polygenic traits. The contents of this Research Topic may serve to identify critical research gaps, and pave the path for future studies.

Obesity, metabolic syndrome (metS) and T2D are well-established to be the most common endocrine disorders with polygenic traits. Nevertheless, the available treatment options may not be successful in the long term as discussed elsewhere (11). Differential expressions of numerous genes are observed in obesity and T2D and miRNAs are involved in transcriptional regulation of target mRNAs (12). Zhang et al. identified upregulation of miR-342-3p as a positive regulator of appetite *via* reduced expression of snap25 gene, which leads to functional impairment of hypothalamic neurons and excess of food intake in a mouse model with fructose induced obesity. A similar study by Yamaguchi et al. identified overexpression of miR-222-3p as a positive regulator of obesity *via* increased adipogenesis. Inhibition of specific miRNAs may serve to address obesity and its complications by promoting novel treatments. Moreover, another novel suggestion to prevent and treat diabetes *via* mesenchymal stromal cells-derived small extracellular vesicles (MSC-sEVs) was reviewed by Li et al. These vesicles contain ncRNA, protein and DNA (13), and the authors report that MSC-sEVs repair or prevent damage from the complications of diabetes through several mechanisms, such as anti-inflammatory effects and reduction of endoplasmic reticulum-related protein stress.

Non-alcoholic fatty liver disease (NAFLD) is a complication of obesity associated with T2D. Several trials have been performed to treat NALFD with variable success (14). A major reason for lack of absolute success with the available treatment is that the cellular and molecular mechanisms leading to NAFLD are still unclear. Xu et al. examined the impact of a lncRNA growth arrest specific 5 (GAS5) on hepatic lipid metabolism in fatty liver in high fat diet-fed obese mice and showed that downregulation of GAS5 led to a series of intracellular events mitigating hepatic lipid metabolism. Therefore, downregulation of GAS5 may be a promising therapeutic option for NAFLD.

Owing to the well-established increase in prevalence of obesity, the distinction between Type 1 Diabetes (T1D) and T2D may be challenging. This is particularly of relevance in some circumstances, especially in Far East countries such as China, where the “thin-phenotype” T2D is more common than the usually expected obese phenotype (15). Lv et al. examined the differential expression of lncRNAs in in peripheral blood leukocytes from patients with T1D, T2D and healthy controls and found four lncRNAs that could be of importance to distinguish T1D and T2D since the treatment differs between the two entities. Further contributing to the diagnostic value of lncRNAs in hyperglycemic states, Yan et al. identified three lncRNAs in patients with hypertriglyceridemia, which could distinguish those with T2D and prediabetes or normoglycemia. The clinical use of these lncRNAs as biomarkers remains to be determined.

In an effort to provide a biologically plausible explanation to the “thin-phenotype” T2D in Far East (15) and South East Asia (16), Sletner et al. examined the association of placental leptin gene DNA methylation with maternal ethnicity, elevated low density lipoprotein-cholesterol and maternal hyperglycemia and found that South Asian ethnicity and gestational diabetes were associated with higher placental leptin gene methylation.

Mir et al. examined the association of miRNAs with dysglycemia in obese patients and found that they were associated with at least one trait of metS. Nevertheless, owing to the relatively low number of the patients enrolled in the study, and the cross sectional nature of the study, further studies are needed to confirm the validity of the differentially expressed miRNAs as biomarkers of metabolically unhealthy obesity.

The utility of miRNAs as biomarkers is not limited to mets components but they can also be of use as a diagnostic marker in microvascular complications of diabetes such as diabetic retinopathy (DR) (17). To this end, Ma et al. performed a meta-analysis investigating the validity of miRNAs as biomarkers of DR, and reported that they can be used with high diagnostic accuracy.

In addition to their potential role as biomarkers, miRNAs may also have a role in adaptive responses to stressors such as hypoglycemia. Ramanjaneya et al. documented that expression levels of miRNAs significantly differed in patients with T2D subjected to insulin induced hypoglycemia versus healthy controls. They showed that blunting of the response to hypoglycemia in patients with T2D with disease of short duration was associated with loss of counter-regulatory response in parallel with the lack of miRNA response.

Exposure to endocrine disruptors at critical times/stages of metabolic programming may also lead to epigenetic changes that may affect phenotype (2). Tawar et al. showed that organochlorine pesticides were associated with endoplasmic reticulum stress markers on adipose tissue samples of patients with T2D *versus* normoglycemic subjects, which further reinforced the concept that endocrine disruptors may be important as contributors to T2D, revealing the persistence of the gene-environment interaction in the etiology of T2D.

The use of miRNAs as biomarkers and/or surrogates of metabolism is not limited to obesity, metS and its complications. Four articles in the current Research Topic describe potential utility of miRNAs as either diagnostic biomarker or follow-up variable in other endocrine disorders: Baloun et al. reported associations of circulating miRNAs with estrogen status and bone metabolism in postmenopausal osteoporosis. Catellani et al. reported changes in circulating levels of miRNAs in patients with growth hormone(GH) deficiency after onset of GH treatment and indicated that the changes in several serum miRNA levels at three months of treatment were associated with the treatment response to GH at one year. Using knock-down or overexpression of the miRNA let-7e-5p in the muscle tissue of orchiectomized(ORX) and androgen treated ORX mice, Okamura et al. suggested that let-7e-5p could be a potential diagnostic marker for muscle atrophy. Galuppini et al. reported potential associations of microRNAs in papillary thyroid cancer with histological variants, which is of clinical significance owing to prognostic implications on follow-up. The available data suggest that miRNAs may be of use in clinical practice in the new era.

Metabolomics is an emerging approach in a systems biology field. Metabolome’s measurement is nowadays frequently implemented to understand pathophysiological processes involved in disease progression as well as to search for new diagnostic or prognostic biomarkers of various organism’s disorders (18). Xia et al. assessed patients with Graves disease and found significant alterations in transfer RNA biosynthesis, amino acids,purine and primidine metabolism. The impact of these changes on the outcome of hyperthyroidism remain to be determined.

In conclusion, we hope that this Research Topic will serve as a point of reference and source of inspiration for researchers and clinicians interested in epigenetic regulation of endocrine disorders. While there has been a massive increase in studies assessing the interrelation of miRNAs, DNA methylation changes and endocrine disruptors with endocrine disorders with polygenic inheritance and/or autoimmune nature, there is a call for further in-depth evaluation of the putatively influential associations of these disorders with ncRNAs other than miRNAs.

## Author contributions

AU drafted the manuscript and agreed to be accountable for all aspects of the work in ensuring that questions related to the accuracy or integrity of any part of the work are appropriately investigated and resolved. All co-authors revised the manuscript for important intellectual content, and approved the final version to be published.

## Acknowledgments

We would like to thank all the contributors to the Research Topic.

## Conflict of interest

The authors declare that the research was conducted in the absence of any commercial or financial relationships that could be construed as a potential conflict of interest.

## Publisher’s note

All claims expressed in this article are solely those of the authors and do not necessarily represent those of their affiliated organizations, or those of the publisher, the editors and the reviewers. Any product that may be evaluated in this article, or claim that may be made by its manufacturer, is not guaranteed or endorsed by the publisher.
